# Genetic and clinical correlates of entosis in pancreatic ductal adenocarcinoma

**DOI:** 10.1038/s41379-020-0549-5

**Published:** 2020-04-29

**Authors:** Akimasa Hayashi, Aslihan Yavas, Caitlin A. McIntyre, Yu-jui Ho, Amanda Erakky, Winston Wong, Anna M. Varghese, Jerry P. Melchor, Michael Overholtzer, Eileen M. O’Reilly, David S. Klimstra, Olca Basturk, Christine A. Iacobuzio-Donahue

**Affiliations:** 1 The David M. Rubenstein Center for Pancreatic Cancer Research, Sloan Kettering Institute, Memorial Sloan Kettering Cancer Center, New York, NY USA; 2 Human Oncology and Pathogenesis Program, Sloan Kettering Institute, Memorial Sloan Kettering Cancer Center, New York, NY USA; 3 Department of Pathology, Memorial Sloan Kettering Cancer Center, New York, NY USA; 4 Cancer Biology and Genetics Program, Sloan Kettering Institute, Memorial Sloan Kettering Cancer Center, New York, NY USA; 5 Department of Medicine, Memorial Sloan Kettering Cancer Center, New York, NY USA; 6 Cell Biology Program, Sloan Kettering Institute, Memorial Sloan Kettering Cancer Center, New York, NY USA

**Keywords:** Pancreatic cancer, Cancer genetics

## Abstract

Entosis is a type of regulated cell death that promotes cancer cell competition. Though several studies have revealed the molecular mechanisms that govern entosis, the clinical and genetic correlates of entosis in human tumors is less well understood. Here we reviewed entotic cell-in-cell (CIC) patterns in a large single institution sequencing cohort (MSK IMPACT clinical sequencing cohort) of more than 1600 human pancreatic ductal adenocarcinoma (PDAC) samples to identify the genetic and clinical correlates of this cellular feature. After case selection, 516 conventional PDACs and 21 ASCs entered this study and ~45,000 HPFs (median 80 HPFs per sample) were reviewed; 549 entotic-CICs were detected through our cohort. We observed that entotic-CIC occurred more frequently in liver metastasis compared with primary in PDAC. Moreover, poorly differentiated adenocarcinoma or adenosquamous carcinoma had more entotic-CIC than well or moderately differentiated adenocarcinoma. With respect to genetic features *TP53* mutations, *KRAS* amplification, and *MYC* amplification were significantly associated with entosis in PDAC tissues. From a clinical standpoint entotic CICs were independently associated with a poor prognosis by multivariate Cox regression analysis when considering all cases or primary PDACs specifically. These results provide a contextual basis for understanding entosis in PDAC, a highly aggressive cancer for which molecular insights are needed to improve survival.

## Introduction

Entosis is a type of regulated cell death that originates from actomyosin-dependent cell-in-cell (CIC) internalization and is executed by lysosomes [1]. Entosis was first discovered as a nonapoptotic cell death process in 2007 [2], and thought to have an advantage for cancer cell survival by promoting cell competition through direct cell–cell interactions unlike other forms of cell death including ferroptosis that occurs at the level of individual cells [3]. Entosis is induced microenvironmentally by glucose starvation [4] and has been associated with *TP53* mutations [5]. Moreover, activated *KRAS* and Rac signaling have been implicated in establishment of winner cell status and in promoting cell death [6].

While studies in vitro have revealed molecular mechanisms of entosis, translational studies exploring the clinicopathological and genetic correlates of entosis in patients are limited. The available data point to entosis as a correlate of aggressive behavior. In rectal cancer, head and neck squamous cell carcinoma (HNSCC) and in lung cancer, a CIC pattern has been associated with poor prognosis [5, 7, 8]. In breast cancer, the CIC pattern is more prevalent in high grade tumors [9]. In pancreatic ductal adenocarcinoma (PDAC), we have recently shown that mutant *TP53* status and/or metabolic related gene expression changes are positively correlated with formation of entotic CIC [10]. However, the extent to which entosis correlates with metastatic propensity remains unknown, as does the extent to which genes other than *TP53* contribute to this phenotypic change. For this reason, we investigated the CIC pattern in PDAC using a large single institution cohort of both primary and metastasis samples for which targeted sequencing data of more than 400 genes are available.

## Materials and methods

### MSK IMPACT clinical sequencing cohort clinical and genetic information

All genetic and clinical information and digital whole slide images were obtained from the MSK IMPACT Clinical Sequencing Cohort database through cBioPortal (version2.2.0) [11]. Genetic information available for each case was based on results of a targeted sequencing panel for up to 468 known and predicted cancer genes as previously described [12]. Treatment information (chemotherapy or chemoradiation therapy) was obtained from an IRB approved and HIPAA compliant RED Cap Database (version 1.0) available through the Center for Pancreatic Cancer Research.

### MSK IMPACT clinical sequencing cohort case selection

**Fig. 1:**
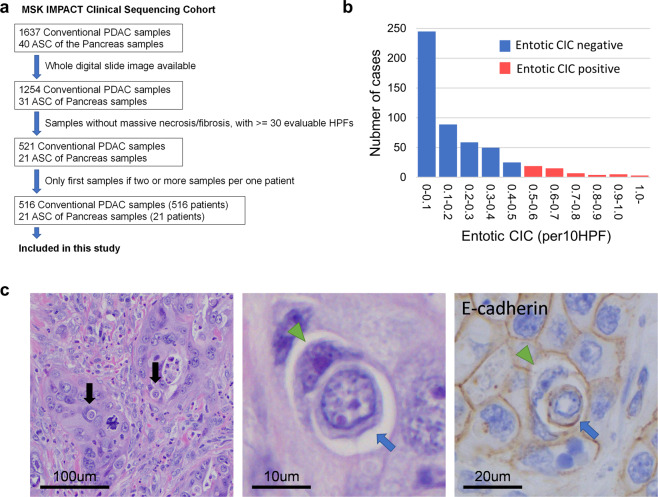
Overview of study set and entotic-CICs. **a** Schematic of sample selection for current study. **b** Distribution of entotic-CIC (per 10 HPFs) in PDACs. **c** Representative histomorphologic and immunohistochemical (E-cadherin) images of entotic-CICs. Entotic-CICs with intervening vacuolar spaces (arrows) were confirmed in low power view. High power view in the middle illustrating a loser cell (blue arrow) engulfed by a winner cell (green arrowhead). E-cadherin confirms entotic-CICs due to the presence of membranous labeling of both the winner and loser cells.

Summary of case selection was shown in Fig. 1a. All genetic and clinical information and digital whole slide images were obtained from the MSK IMPACT Clinical Sequencing Cohort database through cBioPortal (version2.2.0) [11]. Of the PDAC cases, 1637 (conventional or tubular) ductal adenocarcinomas (PDAC) and 40 adenosquamous carcinomas (ASCs) for which both genetic information and digital images were available for study. All hematoxylin and eosin (H&E) digital slides were reviewed by two gastrointestinal pathologists (AH and CAI-D). Samples with massive necrosis and/or degeneration and/or fibrosis, or small biopsy samples with <30 evaluable high-power fields (HPFs) were excluded from this study. In addition, in each sample, tumor regions with degeneration and/or necrosis and/or low tumor content (<10%) were excluded. Finally, a sample set of 537 pancreatic neoplasms (516 conventional PDACs and 21 ASCs) (Fig. 1a) were included in this study.

### Histologic definition of entotic CIC

Entotic CIC structures were defined using H&E images as reported previously [10] as originally proposed by MacKay [5]: cytoplasm of the host cell (winner or engulfing cell), nucleus of the host cell (typically crescent-shaped, binucleate, or multilobular and pushed against the cytoplasmic wall), an intervening vacuolar space surrounding the internalized cell (loser), cytoplasm of internalized cell, and nucleus of internalized cell (often round in shape and located centrally or acentrically). If internalized and/or engulfing cells were undergoing typical mitosis or suspected apoptotic changes they were excluded from analysis. Apoptotic changes were characterized by pyknotic nuclei, nuclear fragmentation, and loss of nuclear detail.

### MSK IMPACT clinical sequencing cohort slide review

Digital whole slides available for the 537 cases selected for study from the MSK IMPACT Clinical Sequencing Cohort were visualized through Smart Slide Viewer (version 1.3.1583). All evaluable tumor regions submitted to the IMPACT sequencing in digital slides were reviewed by two pathologists (AH and CID). A total ~45,000 HPFs (range 30–197 HPFs, median 80 HPFs per sample) were reviewed and all potential or suspected entotic-CICs (~1000 unique images) were captured and reviewed with discussion. Entotic-CICs were quantified by calculating the average number per ten HPFs. Based on this information we divided the cohort into two groups as previously reported [10]. “Entotic-CIC positive” PDACs were defined as those with 0.5 or more entotic CICs per ten HPFs, corresponding to the top ~10% in this study. PDACs not meeting these criteria were defined as “entotic-CIC negative”. Tumor grading (well-, moderately-, poorly differentiated adenocarcinoma) of all cases was determined based on WHO classification [13].

### Immunohistochemistry of E-cadherin

The histologic classification of entosis in 15 representative cases with entotic-CICs from the MSK IMPACT Clinical Sequencing Cohort was validated using immunohistochemistry for E-cadherin [14]. Five micrometer unstained sections were cut from the FFPE blocks of each case, and immunolabeled with a mouse monoclonal antibody against E-cadherin (BD Biosciences, Catalog No. 610181, clone 36/E-Cadherin, 5 ug/ml) according to optimized protocol on a Ventana Benchmark XT autostainer (Ventana Medical Systems Inc.) in the MSK Pathology Core Lab. Appropriate positive and negative controls were included in each run.

### Genetic features of cohort

Using cBioportal as a guide the top ten most frequently mutated genes and the top five most frequent copy number alterations in the cohort of 537 patients were included for correlation to entotic-CICs.

### Autopsy cohort

Matched primary and metastatic tissues from three patients in the Last Wish Program at Memorial Sloan Kettering Cancer Center and 21 patients from the Gastrointestinal Cancer Rapid Medical Donation Program at Johns Hopkins Hospital were used for the validation autopsy cohort. All slides were reviewed by two gastrointestinal pathologists (AH and CID). At least two slides (median eight slides, range 2–12) and 50 HPFs (median 379 HPFs, range 52–1078) for each primary and metastasis site were evaluated for entotic-CICs. Only metastases from the liver, lung, and/or peritoneal cavity were included.

### Ethics statement

This study was approved by the Review Boards of Johns Hopkins School of Medicine and Memorial Sloan Kettering Cancer Center.

### Statistics

All statistics and graphs were performed and made using GraphPad Prism (version 8.2.1) and/or XLSTAT (version 2019.4.2). Each analysis method was described in *Results* or *Figure Legends*. Statistical significance was considered if *P* value is <0.05.

## Results

### Entotic cell in cell structures in PDAC

We identified 539 entotic-CICs within 537 PDAC (516 conventional PDAC and 21 ASC) cases in the MSK IMPACT Clinical Sequencing Cohort. Entotic-CICs per ten HPFs ranged from 0 to 3.06 (median 0.12) per neoplasm (Fig. 1b and c). Per our predefined criteria (see Materials and Methods), 70 of 537 carcinomas (13.0%) correspond to entotic-CIC positive PDACs. All (70/70, 100%) entotic-CIC positive cases had two or more entotic-CICs and more than 80% (59/70 cases, 84.3%) of cases had three or more. Representative CIC patterns (~50 CICs in 15 cases) were confirmed by E-Cadherin IHC that demonstrated membranous labeling of both the winner cell and the cell being engulfed (Fig. 1c).

### Clinical characteristic of entotic-CIC positive PDAC

Entotic-CIC positive status was unrelated to age, gender in univariate analysis of the IMPACT cohort (Table 1). No significant differences were seen for entotic CIC positivity between PDAC and ASC in total cohort (Table 1), although when the analysis was limited to primary tumors only poorly differentiated adenocarcinoma (23 out of 111 samples, 20.7%) or ASC (5/19, 26.3%) had more entotic-CIC positivity than well (2/75, 2.7%) or moderately (10/210, 4.8%) differentiated adenocarcinoma tumors (*P* < 0.0001, the two-sided Fisher’s exact test) (Table 2). For primary surgical resected cases, cases with neoadjuvant chemo- or chemoradiation-therapy had less entotic-CIC (3/69, 4.3%) compared with treatment naïve primary tumors (37/340, 10.9%), but this difference was not statistically significant (*P* = 0.120, the two-sided Fisher’s exact test) (Table 2). No differences were confirmed between lymph node positive and negative cases (Table 2). Entotic-CIC positivity was significantly more prevalent in metastases (30 out of 122 samples, 24.6%) compared with unmatched primary tumors (40/415 samples, 9.6%) (*P* < 0.0001, the two-sided Fisher’s exact test) (Table 1). Among metastatic sites, entotic CIC positive samples most often corresponded to liver metastases (24/69, 34.8%) (Table 3).

**Table 1:** Clinicopathologic characteristics of entotic-CIC positive PDAC.

**Factor**			**Entotic-CIC**			
		**Total**	Positive	Negative	% Positive	*P* Value
Age	>65	317	45	272	14.2%	0.364
	≤65	220	25	195	11.4%	
Gender^a^	Male	299	39	260	13.0%	1.000
	Female	237	31	206	13.1%	
Histology^b^	PDAC	516	65	451	12.6%	0.175
	ASC	21	5	16	23.8%	
Location	Primary	415	40	375	9.6%	**<0.0001**
	Metastasis	122	30	92	24.6%	

The bold value means statistically significant *P* value.

Each *P* value was calculated with the Fisher’s exact test, two-sided.

^a^Gender information was not available for one patient.

^b^*PDAC* Pancreatic ductal adenocarcinoma, *ASC* Adenosquamous carcinoma.

**Table 2:** Primary characteristic of entotic-CIC positive PDAC.

**Factor**			**Entotic-CIC**			
		**Total**	Positive	Negative	% Positive	*P* Value
Tumor grade	Well	75	2	73	2.7%	**< 0.0001**
	Moderately	210	10	200	4.8%	
	Poorly	111	23	88	20.7%	
	ASC^#^	19	5	14	26.3%	
Lymph node metastasis^a^	Positive	290	28	262	9.7%	1.000
	Negative	118	11	107	9.3%	
Neoadjuvant therapy^b^	Yes	69	3	66	4.3%	0.120
	No	340	37	303	10.9%	

The bold value means statistically significant *P* value.

Each *P* value was calculated with the Fisher’s exact test, two-sided.

^a^Lymph node metastasis information is available for 408 surgical resected cases.

^b^Neoadjuvant therapy information is available for 409 surgical resected cases. 48 cases had chemotherapy and 21 cases had chemoradiation therapy.

^#^ASC: adenosquamous carcinoma.

**Table 3:** Metastatic characteristic of entotic-CIC positive PDAC.

Metastasis location		Entotic-CIC		
	**Total**	Positive	Negative	% Positive
Liver	69	24	45	34.8%
Peritoneal cavity	25	2	23	8.0%
Lung	12	2	10	16.7%
Other^a^	16	2	14	12.5%

^a^Others include metastases of lymph nodes (6), soft tissue (2), brain (1), pleura (1), retroperitoneum (1), arm (1), skin (1), umbilicus (1), unknown (2).

**Fig. 2:**
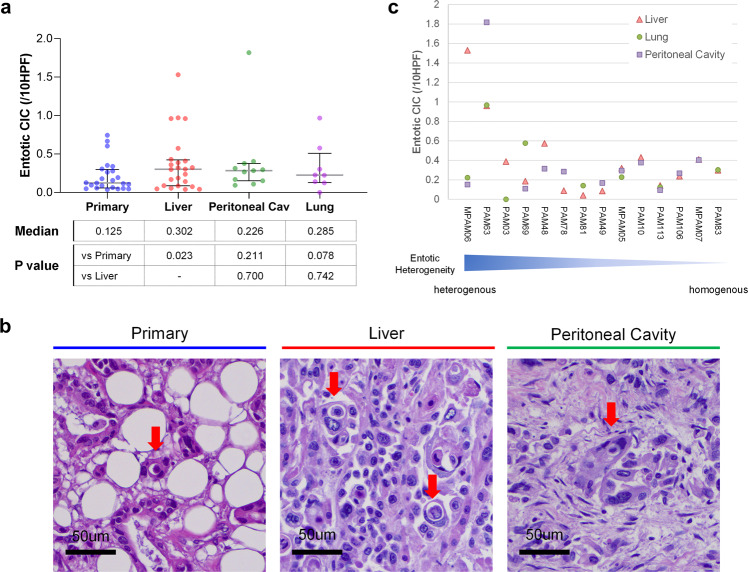
Heterogeneity of entotic-CICs in PDAC. **a** Entotic-CIC (per ten HPFs) in matched primary, and liver, peritoneal cavity and lung metastasis in end-stage PDAC autopsy cohort. **b** Representative histomorphologic images of entotic-CICs in primary and live and peritoneal cavity metastases in autopsy series MPAM06. More than one entotic-CICs were found in one HPF in liver metastasis. **c** Heterogeneity of entotic-CIC among metastatic organs in end-stage PDAC autopsy cohort.

We next reviewed matched primary and liver metastasis samples from an independent cohort of patients who underwent an autopsy. This confirmed that liver metastases had more entotic-CICs than primary carcinomas (*P* = 0.023, the Wilcoxon matched-pair signed rank test, two-sided) (Fig. 2a, b). To determine the extent that different sites of metastasis in the same patient exhibit entotic-CICs we further assessed available lung or peritoneal metastases in these patients. This revealed heterogeneity across the primary tumor and metastases in that entotic-CICs in one site were not indicative of a high number of entotic-CICs in another metastatic site in the same patient, though the degree of heterogeneity varied among cases (Fig. 2a, c).

### Genetic characteristics of entotic-CIC positive PDAC

**Fig. 3:**
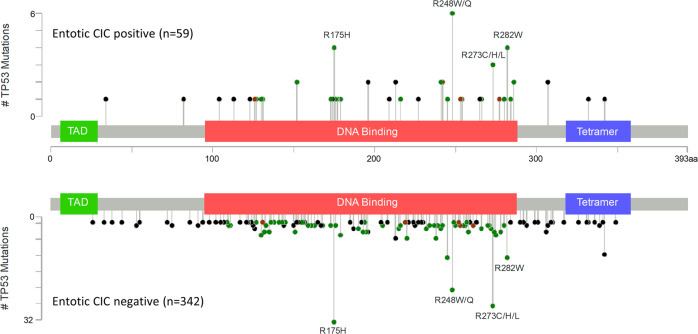
Lolliplot of *TP53* in entotic-CIC positive and negative PDACs. Number of total cases is shown in parentheses.

Entotic-CIC positive PDACs had a higher prevalence of *TP53* mutations (*P* = 0.041, the Fisher’s exact test, two-sided) and *KRAS* and *MYC* amplification (*P* = 0.025 and 0.012, the Fisher’s exact test, two-sided) (Table 4). Deep deletions for *CDKN2A, TP53,* or *SMAD4* were not statistically associated with entotic-CIC positivity (Table 4). To determine if a specific type of *TP53* alteration was associated with the formation of entotic-CICs we compared lollipop plots of the mutational spectrum of *TP53* in entotic-CIC positive vs. negative cases (Fig. 3). We found no difference between the prevalence of hotspot mutations (*P* = 0.331, the Fisher’s exact test, two-sided) nor did we find a difference in the proportion of truncating and non-truncating *TP53* mutations between the two groups (*P* = 1.000, the Fisher’s exact test, two-sided). (Supplementary Information 1). The distribution of *TP53* mutations also did not differ among CIC positive and negative carcinomas among primary and metastatic sites specifically (Supplementary Information 2). While a prior report suggested expression of KRAS-G12V in eating cells is associated with entosis [6], we observed no relationship of a specific *KRAS* hotspot mutation in our cohort (*P* = 0.501, the Fisher’s exact test, two-sided) (Supplementary Information 3).

**Table 4:** Genetic characteristics of entotic-CIC positive PDAC.

**Category**	**Gene**			**Entotic-CIC**			
			**Total**	Positive	Negative	% Positive	***P*** **Value**
Mutation	*KRAS*	Mutated	492	68	424	13.8%	0.102
		WT	45	2	43	4.4%	
	*TP53*	Mutated	399	59	340	14.8%	**0.041**
		WT	138	11	127	8.0%	
	*CDKN2A*	Mutated	137	24	113	17.5%	0.078
		WT	400	46	354	11.5%	
	*SMAD4*	Mutated	113	20	93	17.7%	0.115
		WT	424	50	374	11.8%	
	*CDKN2AP16INK4A*	Mutated	97	15	82	15.5%	0.410
		WT	440	55	385	12.5%	
	*ARID1A*	Mutated	56	10	46	17.9%	0.292
		WT	481	60	421	12.5%	
	*CDKN2AP14ARF*	Mutated	59	10	49	16.9%	0.313
		WT	478	60	418	12.6%	
	*RNF43*	Mutated	29	4	25	13.8%	0.782
		WT	508	66	442	13.0%	
	*KDM6A*	Mutated	28	5	23	17.9%	0.393
		WT	509	65	444	12.8%	
	*APC*	Mutated	25	2	23	8.0%	0.759
		WT	512	68	444	13.3%	
CNV	*MYC*	Amplified	14	5	9	35.7%	**0.025**
		No amplified	523	65	458	12.4%	
	*AKT2*	Amplified	18	6	12	33.3%	0.205
		No amplified	519	64	455	12.3%	
	*CCNE1*	Amplified	10	3	7	30.0%	0.130
		No amplified	527	67	460	12.7%	
	*KRAS*	Amplified	8	4	4	50.0%	**0.012**
		No amplified	529	66	463	12.5%	
	*FGFR1*	Amplified	6	1	5	16.7%	0.569
		No amplified	531	69	462	13.0%	
	*CDKN2A*	Deep deletion	45	6	39	13.3%	1.000
		No deletion	492	64	428	13.0%	
	*CDKN2AP16INK4A*	Deep deletion	44	6	38	13.6%	0.818
		No deletion	493	64	429	13.0%	
	*CDKN2AP14ARF*	Deep deletion	42	6	36	14.3%	0.811
		No deletion	495	64	431	12.9%	
	*CDKN2B*	Deep deletion	40	4	36	10.0%	0.806
		No deletion	497	66	431	13.3%	
	*SMAD4*	Deep deletion	18	2	16	11.1%	1.000
		No deletion	519	68	451	13.1%	

The bold value means statistically significant *P* value.

Each *P* value was calculated with the Fisher’s exact test, two-sided.

### Outcome of entotic-CIC positive PDAC

**Fig. 4:**
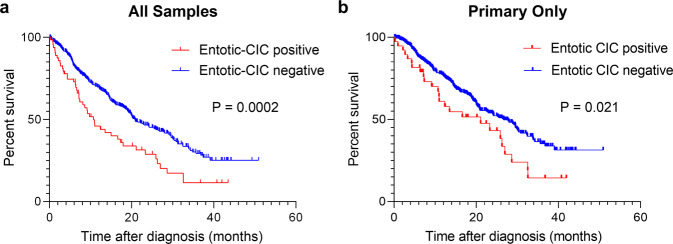
Kaplan–Meier analysis of entotic-CIC positive and negative PDAC. **a** Entotic-CIC positive PDAC (*n* = 65) showed poorer prognosis than negative PDACs (*n* = 434) (*P* = 0.0002, the log-rank (Mantel–Cox) test) in all samples. **b** Entotic-CIC positive primary PDAC (*n* = 39) showed poorer prognosis than negative primary PDACs (*n* = 350) (*P* = 0.021, the log-rank (Mantel–Cox) test).

Kaplan–Meier analysis revealed significant poorer prognosis of entotic-CIC Positive PDAC when considering all cases (*P* = 0.0002, the log-rank test) (Fig. 4a) or primary carcinomas only (*P* = 0.021, the log-rank test) (Fig. 4b). Cox proportional hazards analysis showed positive entotic-CICs, gender, tumor location, and *KRAS* amplification were all associated with a poor prognosis by both univariate and multivariate analyses (Tables 5 and 6). Entotic-CIC positive status was an independent prognostic variable by both univariate (*P* < 0.001, Hazard ratio: 1.813) (Table 5) and multivariate analysis (*P* = 0.014, Hazard ratio: 1.527) (Table 6). This significance identified by multivariate analysis was also confirmed when we use primary carcinomas only (*P* = 0.018, Hazard ratio: 1.668) (Table 7).

**Table 5:** Entotic-CIC in PDAC and patient outcome (univariate analysis).

Factor			*P* value	Hazard ratio (95% confidence interval)
Clinicopathologic	Entotic-CIC	(Positive vs. negative)	**<0.001**	1.813 (1.323–2.484)
	Age	(>65 vs. < =65)	0.853	1.024 (0.8–1.31)
	Gender	(Male vs. female)	**0.010**	1.380 (1.082–1.761)
	Location	(Primary vs. metastasis)	**<0.001**	0.327 (0–0.423)
	Histology	(PDA vs. ASQ)	0.415	0.778 (0.425–1.424)
Genetic	*KRAS*	(Mutated vs. WT)	0.969	0.991 (0.646–1.521)
	*TP53*	(Mutated vs. WT)	0.173	1.216 (0.918–1.611)
	*CDKN2A*	(Mutated vs. WT)	0.094	1.259 (0.962–1.649)
	*SMAD4*	(Mutated vs. WT)	0.976	0.996 (0.746–1.329)
	*CDKN2AP16INK4A*	(Mutated vs. WT)	0.384	1.141 (0.848–1.534)
	*ARID1A*	(Mutated vs. WT)	0.395	1.179 (0.807–1.725)
	*CDKN2AP14ARF*	(Mutated vs. WT)	0.310	1.199 (0.844–1.703)
	*RNF43*	(Mutated vs. WT)	0.382	0.772 (0.433–1.378)
	*KDM6A*	(Mutated vs. WT)	0.828	1.062 (0.619–1.82)
	*APC*	(Mutated vs. WT)	0.166	1.464 (0.854–2.51)
CNA alteration	*MYC*	(Amplified vs. not amplified)	0.883	0.941 (0.419–2.115)
	*AKT2*	(Amplified vs. not amplified)	0.399	1.297 (0.709–2.371)
	*CCNE1*	(Amplified vs. not amplified)	0.503	0.678 (0.217–2.116)
	*KRAS*	(Amplified vs. not amplified)	**<0.001**	5.480 (2.419–12.416)
	*FGFR1*	(Amplified vs. not amplified)	0.117	2.490 (0.796–7.788)
	*CDKN2A*	(Deep deletion vs. no deletion)	**0.010**	1.631 (1.122–2.372)
	*CDKN2AP16INK4A*	(Deep deletion vs. no deletion)	**0.019**	1.575 (1.077–2.303)
	*CDKN2AP14ARF*	(Deep deletion vs. no deletion)	**0.016**	1.604 (1.091–2.358)
	*CDKN2B*	(Deep deletion vs. no deletion)	0.216	1.291 (0.862–1.934)
	*SMAD4*	(Deep deletion vs. no deletion)	0.242	0.639 (0.302–1.353)

The bold value means statistically significant *P* value.

**Table 6:** Multivariate analysis of entotic-CIC in PDAC and patient.

Factor		*P* value	Hazard ratio (95% CI)
Entotic-CIC	(Present vs. absent)	**0.014**	1.527 (1.090–2.138)
Gender	(Male vs. female)	**0.005**	1.431 (1.115–1.836)
Location	(Primary vs. metastasis)	**<0.001**	0.360 (0–0.474)
TP53	(Mutated vs. WT)	0.328	1.153 (0.866–1.535)
CDKN2A	(Mutated vs. WT)	0.217	1.197 (0.900–1.592)
APC	(Mutated vs. WT)	0.409	1.184 (0.792–1.771)
KRAS	(Amplified vs. not amplified)	**0.020**	2.742 (1.173–6.408)
FGFR1	(Amplified vs. not amplified)	0.944	0.959 (0.295–3.119)
CDKN2A	(Deep deletion vs. no deletion)	0.245	1.382 (0.801–2.386)

**Table 7:** Multivariate analysis of entotic-CIC in PDAC (Primary Only) and patient.

Factor		*P* value	Hazard ratio (95% CI)
Enototic-CIC	(Present vs. absent)	**0.018**	1.668 (1.092–2.548)
Gender	(Male vs. female)	0.181	1.225 (0.910–1.651)
TP53	(Mutated vs. WT)	0.060	1.435 (0.986–2.090)
CDKN2A	(Mutated vs. WT)	0.254	1.217 (0.868–1.708)
APC	(Mutated vs. WT)	0.106	1.825 (0.881–3.781)
KRAS	(Amplified vs. not amplified)	**0.010**	8.242 (1.658–40.974)
FGFR1	(Amplified vs. not amplified)	0.384	2.420 (0.331–17.674)
CDKN2A	(Deep deletion vs. no deletion)	0.415	0.711 (0.314–1.613)

## Discussion

We find that entosis in PDAC is a correlate of aggressive biology and an independent prognostic factor by multivariate analysis. Moreover, metastases appear to have a greater number of entotic-CICs than primary tumors. These findings not only validate prior reports in rectal, head, and neck and breast cancer and but provide a deeper understanding of the clinicopathologic relevance of entosis in PDAC.

Our finding that entotic-CICs are more prevalent in poorly differentiated PDAC parallels prior observations in breast cancer [9]. We extend this observation by showing that pancreatic ASC also have a high number of entotic-CICs. This is consistent with our prior reports that squamous features in PDAC arise in the context of clonal evolution and that ASC has more entotic-CICs than poorly differentiated carcinoma [10]. Furthermore, our finding of an association of *TP53* mutations with entotic-CIC parallels prior reports in lung cancer [5]. However, we now clarify the lack of an association with *TP53* hotspot mutations. While the mechanisms by which TP53 alterations contribute to entosis is unknown, one putative molecular mechanism is that loss of TP53 or delta-133TP53 expression increases extracellular ATP release and the consequent activation of purinergic P2Y2 receptors, which induces cell engulfment [15]. Alternatively, TP53 activation may cause upregulation of Rnd3, which inhibits ROCK1 or RhoA activities directly or indirectly, leading to polarized activation of RhoA-actomyosin, which drives cell internalization to form CIC structures [16].

The reason for an increased prevalence of entotic-CICs in metastatic PDAC, specifically liver metastases, also remains to be determined. However, we expect that the mechanisms are multifactorial. For example, a recent report showed *TP53* inactivation and *KRAS* amplification are more frequent in metastasis [17]. Our data confirms this phenomenon because of eight *KRAS* amplified samples, six were liver metastases and only two were primary carcinomas (*P* < 0.0001, the chi square test with Yates correction). While PDAC metastases does not appear to have a genetic basis [18], subclonal evolution at the genetic level with respect to gene dosages may select for the entotic phenotype. We suspect the tumor microenvironment may also play a role. For example, while primary tumors are rich in stromal cells and poorly vascularized, the liver is by contrast a highly vascular tissue with little stromal response until the metastases exceeds a critical mass of cells [19]. Thus, it is conceivable that changes in the microenvironment and available nutrients may select for the entotic phenotype within specific genetic contexts.

Poor prognosis in entotic-CIC high tumors that we report here have been previously reported in lung adenocarcinoma [5], HNSCC [7, 8], rectal and anal carcinoma [8]. However, given that only in HNSCC has entotic-CIC been shown to be an independent prognostic factor in HNSCC [7], this is the first demonstration of entotic -IC as an independent prognostic factor in adenocarcinoma. While we cannot totally exclude the possibility of simple dimensional overlap or emperipolesis [20] in tumor cells reported previously, we believe that our simple methods to evaluate entotic-CIC can be applied to practical diagnostic pathology. We also expect that our findings will also have implications for understanding entotic-CIC in other solid tumor types.

## Supplementary information

Supplementary Information 1

Supplemenatry Information 2

Supplementary Information 3
