# Supplementary material for: Genetic and clinical correlates of entosis in pancreatic ductal adenocarcinoma
Source: Mod Pathol. 2020 Apr 29;33(9):1822–31. doi: 10.1038/s41379-020-0549-5 (PMC7452867; doi:10.1038/s41379-020-0549-5)
Supplement: Supplementary file 1 — Supplementary Information 1 [file 41379_2020_549_MOESM1_ESM.pdf]

Supplementary Information 1. *TP53* Hot Spot Mutation in Entotic-CIC Positive PDAC

***TP53* mutation**		**Total**	**Entotic-CIC**			
			Positive	Negative	% Positive	**P-Value**
Hotspot	R175H	39	4	35	10.3%	0.331
	R248W/Q	28	6	22	21.4%	
	R273C/H/L/S	30	3	27	10.0%	
	R282W	16	4	12	25.0%	
Truncating		135	20	115	14.8%	1.000
Non-trauncating		265	39	226	14.7%	

P-value was calculated with Fisher's exact test, two-sided.
