# Supplementary material for: Genetic and clinical correlates of entosis in pancreatic ductal adenocarcinoma
Source: Mod Pathol. 2020 Apr 29;33(9):1822–31. doi: 10.1038/s41379-020-0549-5 (PMC7452867; doi:10.1038/s41379-020-0549-5)
Supplement: Supplementary file 2 — Supplemenatry Information 2 [file 41379_2020_549_MOESM2_ESM.pdf]

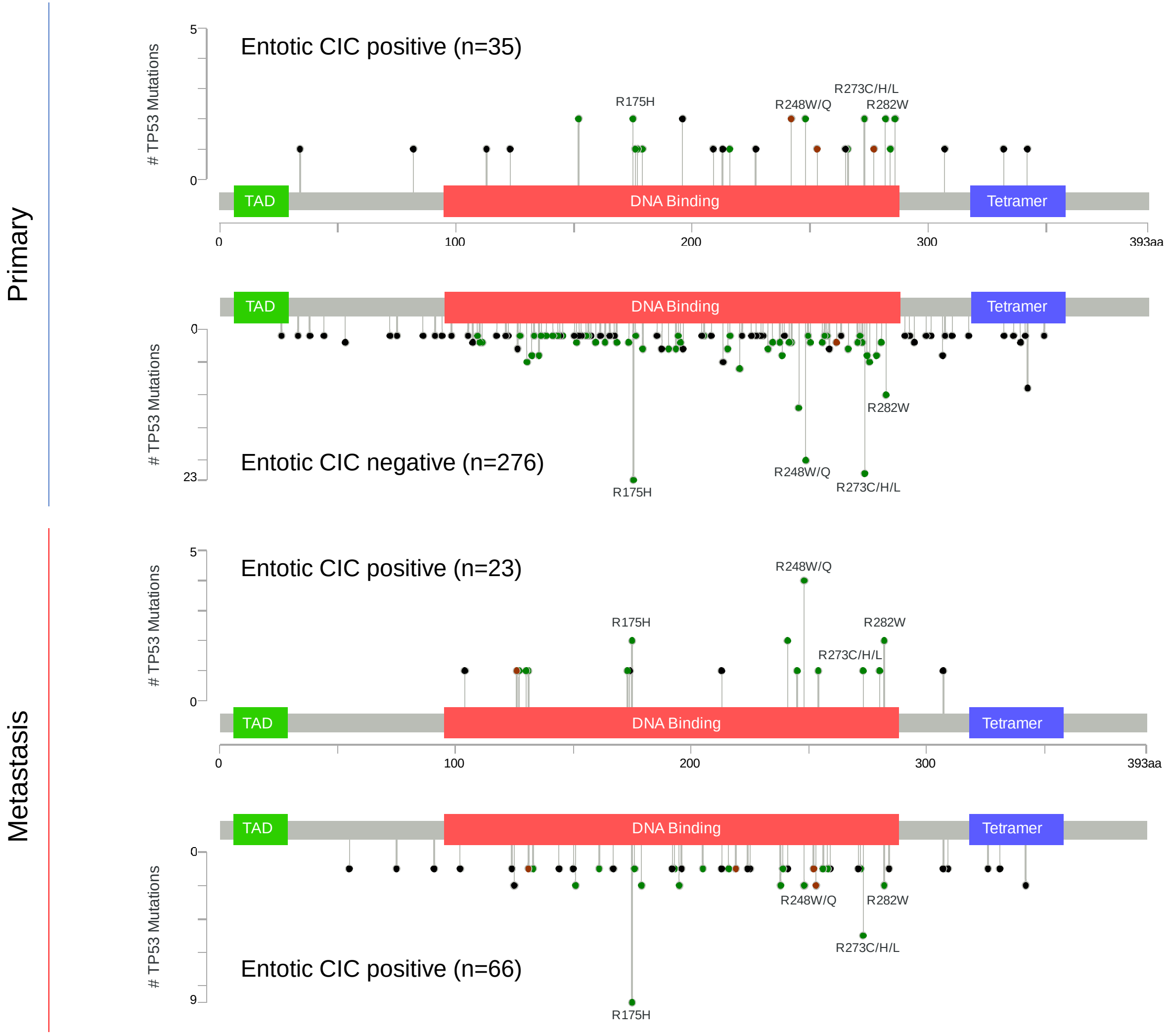


## Supplementary Information 2

Lolliplot of TP53 in entotic CIC positive and negative PDACs for primary and metastasis.
