# Supplementary material for: Genetic and clinical correlates of entosis in pancreatic ductal adenocarcinoma
Source: Mod Pathol. 2020 Apr 29;33(9):1822–31. doi: 10.1038/s41379-020-0549-5 (PMC7452867; doi:10.1038/s41379-020-0549-5)
Supplement: Supplementary file 3 — Supplementary Information 3 [file 41379_2020_549_MOESM3_ESM.pdf]

Supplementary Information 3. *KRAS* Hot Spot Mutation in Entotic-CIC Positive PDAC

***KRAS* mutation**		**Total**	**Entotic-CIC**			**P-Value**
			Positive	Negative	% Positive	
Hotspot	G12D	213	28	185	13.1%	0.501
	G12V	166	27	139	16.3%	
Others		113	13	100	11.5%	

P-value was calculated with Fisher's exact test, two-sided.
